# Business Environment and Entrepreneurial Motivations of Urban Students

**DOI:** 10.3389/fpsyg.2020.01483

**Published:** 2020-07-28

**Authors:** Feixia Wu, Chuanyu Mao

**Affiliations:** College of Entrepreneurship, College of Oujiang, Wenzhou University, Wenzhou, China

**Keywords:** environmental perception, urban entrepreneurial environment, college students, entrepreneurial motivation, influencing factors

## Abstract

College students’ perceptions of entrepreneurial environments exert significant influence on their entrepreneurial motivations. With China’s increased focus on entrepreneurship and innovation, an increasing number of college students have embarked on a journey of self-employment and entrepreneurship. Due to disparities in the urban entrepreneurial environments, however, their entrepreneurial ventures in urban spaces are characterized by extreme phenomena such as entrepreneurial clusters and human capital flight, thus constraining economic development. This study sampled 244 college students from Wenzhou, China, and analyzed their opinions on the relationship between their perceptions of the entrepreneurial environment and associated motivations. We concluded that college students’ entrepreneurial motivations are significantly and positively affected by their perception of socio-economic conditions, education and training, and availability of financial and non-financial support, and insignificantly affected by their perceptions of the favorability of government policies as related to entrepreneurial development.

## Introduction

At present, the paradigm of global economic growth is undergoing a radical transformation in which entrepreneurial activities are gradually gaining prominence as a new driver of economic growth. As the engine and a major player in global economic growth, China contributed to one-third of the world’s aggregate economic growth in 2017 (People’s Daily, 2017). In reality, however, the overall development of the Chinese national economy is hampered by enormous disparities among entrepreneurial activities in different cities.

The local economy, be it at a national, regional, or municipal level, is closely and positively correlated with the vitality of its entrepreneurial ecosystem (Guo et al., 2006). Although the socio-economic issues faced by cities vary greatly, all can be ultimately attributed to the entrepreneurial environment in each city (Dang and Wei, 2011). As of 2018, Beijing, Shanghai, and Guangzhou were among the top-ranked Chinese cities in terms of their innovative and entrepreneurial environments (Tuspark Research Institute for Innovation, 2018), collectively accounting for 55% of the top 100 enterprises in terms of innovation and entrepreneurial capabilities (Chen, 2019). The clustering of outstanding innovative and entrepreneurial talents as well as the agglomeration of high- and new-technology enterprises have remained noticeable across first-tier cities like Beijing, Shanghai, and Guangdong (Chen, 2019). These trends are accompanied by underlying imbalances in human capital distribution and enterprise structure, which have limited the overall economic development of China. While college students are reluctant to develop a career in third- and fourth-tier cities, small and medium-sized enterprises in these cities have the most pressing need for talent. Wenzhou, despite having one of the most vibrant private enterprise ecosystems among Chinese cities, is nonetheless faced with an alarming talent shortage. According to a survey report titled “Addressing the One-Way Exodus of Wenzhou-born College Students with Utmost Priority” (Research Office of Wenzhou. Municipal People’s Government, 2012),Wenzhou had recorded an annual outflow of nearly 15,000 local college students who left the city for entrepreneurial ventures elsewhere; the outflow rate of workers stood at approximately 36% (Wang, 2012). In recent years, as a result of this brain drain, Wenzhou’s economic development has become vulnerable.

It is reasonable to conclude that such clustering or human capital flight and the urban entrepreneurial environment are closely associated with individuals’ entrepreneurial motivations. Thus, a growing group of scholars have directed their attention to pioneering and transforming the urban entrepreneurial environment to stimulate entrepreneurial activities and, in turn, accelerate regional economic development. For instance, Castano et al. (2016) found that individuals’ perceptions of the entrepreneurial environment exert an important influence on their entrepreneurial behaviors and that creating a favorable entrepreneurial environment can encourage individuals’ entrepreneurial intentions.

In 2020, China’s workforce is projected to gain an estimated 100 million young workers (Wang, 2011). The Chinese government is striving to alleviate the ever-mounting two-fold pressure from growing unemployment and imbalance in regional economies by proactively launching related policies and improving the entrepreneurial environment to stimulate college students’ entrepreneurial motivations. Nevertheless, substantial inter-regional disparities in the development of entrepreneurial environments have severely restricted the cultivation of college students’ entrepreneurial motivations and their initiation of entrepreneurial activities. Leveraging the behavioral influence of the positive factors of environmental perception effectively and avoiding or improving some of the negative factors involved in a scientifically reasonable manner are of great significance to nurturing entrepreneurial consciousness and building a strong entrepreneurial motivation among college students, thus promoting the effective initiation of urban entrepreneurial activities (Li and Niu, 2013; Wu et al., 2019).

Wenzhou has one of the most vibrant private enterprise ecosystems among Chinese cities. Selecting the city as an example, this study investigated college students’ perceptions of the city’s entrepreneurial environment and analyzed the corresponding effects on their entrepreneurial motivations, thereby identifying the key factors influencing their initiation of entrepreneurial activities. The findings of this study provide local Chinese governments with important references for improving urban entrepreneurial environments and encouraging college students to initiate entrepreneurial activities.

## Literature Review and Hypotheses Development

### Literature Review

#### Entrepreneurial Motivation

Entrepreneurial motivation is regarded as the first step in instigating entrepreneurial activities, and research in this regard can deepen the understanding of entrepreneurial cognition and behavioral patterns. The implications of entrepreneurial motivation have previously been interpreted by scholars from a variety of perspectives. Olson and Bosserman (1984) identified entrepreneurial motivation as the drive that encourages individuals who already possess the basic conditions for entrepreneurship and considerable entrepreneurial abilities to start a business. Carsrud and Brännback (2011) argued that entrepreneurial motivation is the spark that transforms entrepreneurial cognition and intentions into actual entrepreneurial actions. Zolin et al. (2013) highlighted that entrepreneurial motivation is an entrepreneur’s intention to realize an enterprise’s goals and vision and determines the direction and scale of corporate development.

Previous scholars have studied the various factors that could lead to entrepreneurial motivation and the underlying influence mechanisms involved from the standpoints of personality traits and external environment. Duan et al. (2012) argued that entrepreneurial motivation could be understood as a psychological disposition or inner drive that fuels individuals’ entrepreneurial ventures; it is a special state of mind in which, under environmental influence, individuals translate their entrepreneurial intentions into real actions. Boyd and Vozikis (1994) believed that entrepreneurial motivation is influenced by self-efficacy, Locke and Latham (2002) put forward a theory that entrepreneurs’ behavioral traits can be affected by goals, and Shane et al. (2003) discovered that entrepreneurs’ personal characteristics play a fundamental role in their entrepreneurial decision-making.

Extending beyond the outcome of an entrepreneur’s own personal actions, entrepreneurship is influenced by a variety of external environmental factors such as economic conditions and government laws and regulations. Yoon (2012) found that college students’ entrepreneurship motivations are positively affected by four factors, namely, entrepreneurship education, desire for self-achievement, interpersonal network, and social recognition. Bartha et al. (2019) studied college students’ entrepreneurial motivation in Central and Eastern European countries and established five influencing factors: social mission, customer focus, competition/market focus, individual goals, and collective/community goals.

#### Theory of Planned Behavior

The theory of planned behavior (TPB) is an important theory regarding the attitude–behavior relationship; it was proposed by Ajzen (1991) and was derived from the multi-attribute attitude and reasoned action theories. Applied mainly to explain and predict various behaviors, it facilitates the understanding of how humans change their behavioral patterns. Theory of planned behavior holds that one’s behavioral intentions are indicative of his/her motivation to perform a certain behavior and that these intentions can influence behaviors by activating motivational factors. Intentions are determined by one or a combination of the following factors: attitude, subject norms, and perceived behavioral control. With the ongoing expansion of this line of research, many scholars have applied TPB to discussing the notion of entrepreneurial motivation. For example, Kibler (2013) drew on this theory to explore the relationship between an entrepreneur’s individual characteristics, regional characteristics, and the factors influencing entrepreneurial motivation. Aloulou (2016) sampled 177 business students to test the TPB model, revealing that the antecedents of TPB could significantly explain entrepreneurial motivations.

#### Perception of Entrepreneurial Environment and Motivation

As entrepreneurial activities have become a focal point of academic research, a number of experts and scholars have isolated and studied the environmental factors that significantly influence entrepreneurship. Ahmad and Xavier (2012) believed that the entrepreneurial environment refers to a combination of factors that play a role in entrepreneurship and entrepreneurial activities. Bernhofer and Li (2014) stated that the entrepreneurial environment encompasses the cultural, economic, and political environments and that individuals have different motivations in different environmental backgrounds. Nam and Hwansoo (2019) adopted an external perspective and defined the entrepreneurial environment as the sum of the legal and institutional environment, market environment, financial environment, and entrepreneurial infrastructure, among other aspects.

Many researchers who have analyzed the factors influencing entrepreneurial motivation from the viewpoint of the entrepreneurial environment considered the external environment to be an objective condition for entrepreneurship. Suzuki et al. (2002) believed that entrepreneurial motivation is the product of environmental and individual variables and that it is influenced by managerial skills, managerial resources, market conditions, business culture, and government support. Taormina (2007) conducted an empirical analysis of the relationship between entrepreneurial motivation and the external entrepreneurial environment and pointed out that the former is influenced by achievement motivation, optimism toward life, and social networking. A study conducted by Gohmann (2010) indicated that institutions, particularly managerial institutions, exert greater influence on latent entrepreneurs; individuals are more willing to start a business as the economic freedom of a country increases. Welter (2011) stated that entrepreneurs are influenced by factors such as financial capital, information, emotional understanding, and encouragement. Few researchers have explored the relationship between the perception of the entrepreneurial environment and individuals’ entrepreneurial motivations. For instance, Yao et al. (2016) discovered that college students’ entrepreneurial motivations are affected by their perceptions of the socio-economic environment. Nam and Hwansoo (2019) indicated that entrepreneurs’ attitudes and motivations are significantly affected by their perceptions of the entrepreneurial environment. Zhao et al. (2019) believed that college students evaluate whether the perceived environment is conducive to entrepreneurial activities when contemplating the decision to start a business; their perceptions of a favorable entrepreneurial environment engender an inner drive that encourages entrepreneurship.

Overall, existing studies of college students’ entrepreneurial environment have mostly concentrated on five dimensions: government policies, socio-economic conditions, education and training, financial support, and non-financial support (Table 1). Studies in relation to other dimensions of the entrepreneurial environment have been less focused, spanning areas such as entrepreneurial and managerial skills, socio-cultural environment, market environment, and social networking. Hence, we selected government policies, socio-economic conditions, education and training, and availability of financial and non-financial support as the research dimensions of this study and unraveled their effects on entrepreneurial motivations from a self-perception standpoint.

**TABLE 1:** Environmental factors influencing entrepreneurial motivation.

Influencing factor	Definition	References
Government policies	Preferential measures for entrepreneurs offered by government departments in taxation, facilitation of approval processes, and optimization of entrepreneurial institutions	Gnyawali and Fogged, 1994; Garcia-Cabrera et al., 2018; Nam and Hwansoo, 2019
Socio-economic conditions	Level of development of the local economy, industrial structure, and urban construction; infrastructure such as transport, water supply, and electricity supply; and regional entrepreneurial culture	Davidsson and Honig, 2003; Liñán and Santos, 2007; Gathungu and Baariu, 2018; Martínez-Fierro et al., 2020
Education and training	Innovation and entrepreneurship education, related activities, and capacity-building training attended by entrepreneurs during their studies	Turker and Selcuk, 2009; Sirbu et al., 2015; Bazan et al., 2020
Financial support	Financial support obtained by entrepreneurs in sources of financing, interest-free loans, and start-up funds	Kristiansen and Indarti, 2004; Welter, 2011; Yao et al., 2016
Non-financial support	Assistance received by entrepreneurs in expert guidance, development opportunities, information, and resources	Djankov et al., 2006; Schwartz, 2009; Gathungu and Baariu, 2018

### Research Hypotheses

According to the TPB, college students are highly responsive to perceptions of entrepreneurial opportunities and, in such favorable conditions, are ready to embark on new ventures. They are more inclined to pursue entrepreneurship as a career path when there is stronger environmental support for entrepreneurial behaviors (Liñán and Santos, 2007). Having said this, the entrepreneurial environment has been perennially available to individuals. The perceived entrepreneurial environment is a subjective construct that translates into entrepreneurial motivation only when it is perceived as favorable by college students. Based on the notion of utility maximization, college students’ perceptions of the entrepreneurial environment have a positive effect on their entrepreneurial motivations (Zhao et al., 2019). If this perception is strong, they are likely to consider entrepreneurship to be a feasible option and pursue it as a means to ensure the continuation and accumulation of existing utility. This, in turn, reinforces their entrepreneurial engagement motivations.

Government policies include policy measures, laws, and regulations launched by government departments in taxation, market access, and other relevant aspects. Stevenson and Lundstrom (2001) suggested that, when entrepreneurs perceive that the government’s entrepreneurship policies facilitate the creation of more business opportunities and a better market environment, their entrepreneurial confidence and, by extension, their likelihood of engaging in entrepreneurial activities increase. Government policies that are favorable to entrepreneurship give rise to more entrepreneurial opportunities and promote the realization of entrepreneurial behaviors (Wu et al., 2018). While policy support offers college students a certain level of protection, preferential entrepreneurship policies can incentivize more latent entrepreneurs (Yao et al., 2016). Nam and Hwansoo (2019) found that the accessibility of government policies affects entrepreneurs’ attitudes and behaviors. Based on the above viewpoints, we hypothesized the following.

H1: College students’ perceptions of government policies positively affect their entrepreneurial motivations.

As latent entrepreneurs with the strongest innovation capability among younger generations, college students develop a stronger desire for entrepreneurial engagement once they realize that economic development is advancing in the direction of innovation (Davidsson and Honig, 2003). Liñán and Santos (2007) pointed out that entrepreneurial motivation is also affected by social values and beliefs regarding entrepreneurship. Obschonka et al. (2012) argued that social culture serves as a key determinant of college students’ entrepreneurial motivations and that successful role models stimulate their entrepreneurial beliefs. Martínez-Fierro et al. (2020) found that economic development has a substantial impact on entrepreneurial motivation. Therefore, with regard to socio-economic conditions and entrepreneurial motivation, we hypothesized the following.

H2: College students’ perceptions of socio-economic conditions positively affect their entrepreneurial motivations.

Education and training, as indicated in a number of previous studies, constitute a crucial motivational factor for college students’ entrepreneurial engagements (Wu et al., 2020). Sirbu et al. (2015) revealed that college students have a stronger command of entrepreneurial and managerial skills after receiving entrepreneurship education and training. They become more sensitive to the environment and more sensible of the new entrepreneurial opportunities arising from environmental changes, while also being able to better seize these opportunities. Wu et al. (2018), Hou et al. (2019), and Wu and Song (2019) revealed that students’ motivations to undertake entrepreneurial activities are affected by entrepreneurship education. Bazan et al. (2020) posited that entrepreneurship education affects college students’ entrepreneurial motivations significantly. Hence, we hypothesized the following.

H3: College students’ perceptions of education and training positively affect their entrepreneurial motivations.

Access to capital is one of the biggest obstacles faced by college entrepreneurs. Welter (2011) believed that college students’ entrepreneurial ventures are influenced by financial capital availability due to the inaccessibility of financial support such as project grants or microfinance. According to Chen (2013), the problem of start-up capital availability, including access to low-interest loans, start-up funds, and the diversity of financing, remains as one of the top concerns among college entrepreneurs. When students perceive that the capital at hand is insufficient for starting a business, they are reluctant to attempt entrepreneurial ventures. As college students do not generally generate income, they usually have insufficient start-up capital and find entrepreneurship more difficult than any other group (Yao et al., 2016). Based on the above viewpoints, we hypothesized the following.

H4: College students’ perceptions of financial support availability positively affect their entrepreneurial motivations.

Due to lack of information and resource support, entrepreneurship poses a greater challenge to college students than to any other group. This makes access to non-financial support all the more important (Gathungu and Baariu, 2018; Yuan et al., 2019). Schwartz (2009) found that undertaking entrepreneurial practices in incubator facilities can arouse college students’ entrepreneurial passions and interests. Yao et al. (2016) discovered that college students’ entrepreneurial motivations are affected by the accessibility of social networks, information, and capital and that support in social networks, in particular, enhances their entrepreneurial confidence. Hence, we hypothesized the following.

H5: College students’ perceptions of availability of non-financial support positively affect their entrepreneurial motivations.

**FIGURE 1:**
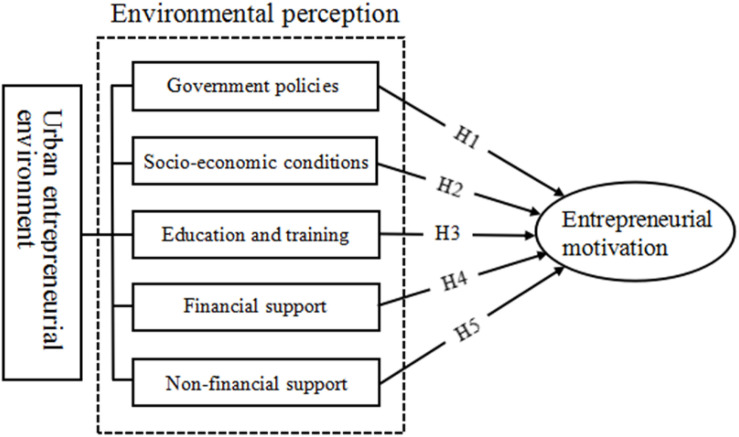
Model of the effects of the perception of entrepreneurial environment on entrepreneurial Motivation among college students.

Based on the above theoretical underpinnings and hypotheses, we constructed a model of the effects of entrepreneurial environment perceptions on entrepreneurial motivations, as shown in Figure 1.

## Research Methods and Design

### Measurement of Variables

To ensure the validity of the questionnaire survey used in this study, we drew on well-developed and already-validated indicator systems from previous literature, used them as a basis for scale development, and made modifications according to the characteristics of college students. After the initial draft was completed, it was distributed to 30 college students for literal validity checks, pre-tests, and final revisions. Eventually, a questionnaire survey pertaining to the model of this study was developed. It comprised basic respondent information and measured respondent attitudes toward six variables: government policies, socio-economic conditions, education and training, financial support, non-financial support, and entrepreneurial motivation. A total of 37 measurement items were involved (Table 2). The statements were scored on a 5-point Likert scale, with 1 representing “completely disagree” and 5 representing “completely agree.”

**TABLE 2:** Measurement items design.

Variable	Item	Item design	References
Government policies	GP1	Government offers preferential tax policies	Gnyawali and Fogged, 1994; Ma, 2014
	GP2	Registration and approval procedures are simple and convenient	
	GP3	Publicity and implementation of relevant policies are widespread	
	GP4	Government offers entrepreneurial counseling services for college students	
	GP5	Well-developed institutions governing entrepreneurial behaviors	
Socio-economic conditions	SEC1	Local economic growth is fast paced	Gnyawali and Fogged, 1994; Ma, 2014
	SEC2	I am influenced by successful entrepreneurial role models	
	SEC3	The city is home to many successful entrepreneurs	
	SEC4	Local culture encourages innovation and risk-taking	
	SEC5	Family members are supportive of entrepreneurial ventures	
Education and training	ET1	I once attended a systematic entrepreneurship education program	Gnyawali and Fogged, 1994; Ma, 2014
	ET2	I often participate in business plan competitions	
	ET3	I am highly experienced in social practices	
	ET4	I often receive entrepreneurial training and guidance	
	ET5	There is a rich entrepreneurial atmosphere in the university	
Financial support	FS1	There are alternative finance sources for student entrepreneurs	Gnyawali and Fogged, 1994; Ma, 2014
	FS2	Low-interest loans offered by banks are accessible	
	FS3	Start-up funds offered by the school/government are accessible	
	FS4	There is fierce competition among local financial institutions	
	FS5	A variety of loan guarantee options are available	
Non-financial support	NFS1	Well-developed local information and communications channels	Gnyawali and Fogged, 1994; Ma, 2014
	NFS2	Research and development is supported by the government	
	NFS3	Government procurement programs are open to small businesses	
	NFS4	The entrepreneurial network is resourceful	
	NFS5	Local incubator facilities are available	
Entrepreneurial motivation	EM1	My career goal is to become an entrepreneur	Suzuki et al., 2002; Liñán and Santos, 2007; Ma, 2014
	EM2	Entrepreneurship can improve my family’s economic conditions	
	EM3	Entrepreneurship can ease the pressure of unemployment	
	EM4	I want to attempt entrepreneurship due to favorable state policies	
	EM5	I am influenced by a relative who has started a business, or other entrepreneurs	
	EM6	Entrepreneurship can elevate my social status	
	EM7	Entrepreneurship allows me to realize my worth and contribute to the society	
	EM8	Entrepreneurship allows me to improve and perfect myself	
	EM9	The school has a rich and favorable entrepreneurial atmosphere, where many of my peers have started their own businesses	
	EM10	I choose to be an entrepreneur because my family can offer me support in funding or human capital	
	EM11	Accumulation of entrepreneurial experiences as a student benefits future employment	
	EM12	Accumulation of entrepreneurial experiences as a student drives me to continue my entrepreneurial career after graduation	

It is also worth noteworthy that their decision-making was directly shaped by their subjective judgments of the environment, despite that individuals were situated in an objective, specific, and real-life environment. Influencing factors in the entrepreneurial environment were thus measured mainly based on the perceptions of the research subjects.

### Data Collection

This study selected current students attending seven higher education institutions in Wenzhou as the research subject. As the birthplace of China’s private economy, Wenzhou boasts a rich entrepreneurial culture with an abundance of entrepreneurial practices, which exert a subtle influence, whether directly or indirectly, on local college students. Furthermore, higher education institutions in Wenzhou give priority to entrepreneurship education and offer a diverse repertoire of entrepreneurship-related educational programs and hands-on activities.

For this study, we adopted an online survey tool called Wenjuanxing^1^ and retrieved a total of 261 completed questionnaires between July and October 2017. After screening, 244 valid copies were returned, representing a valid response rate of 93.49%. A statistical analysis was conducted on the sample data (Table 3). The respondent sample comprised 140 females (57.38%) and 104 males (42.62%). In terms of the level of education, respondents at the undergraduate level comprised 95.49% of the total sample. The most common academic disciplines were economics and management, followed by sciences and engineering, and then humanities and social sciences. Most of the respondents fell into the age group of 20–25 years old, which was fitting for the current study on college students.

**TABLE 3:** Descriptive statistics of the sample.

Variable	Item	Frequency	Percentage
Gender	Male	104	42.62
	Female	140	57.38
Age	Below 20	97	39.75
	20–25	138	56.56
	26–30	7	2.87
	Above 30	2	0.82
Level of education	Junior college	10	4.1
	Undergraduate	233	95.49
	Master’s	1	0.41
Academic discipline	Sciences and engineering	70	28.69
	Economics and management	77	31.56
	Humanities and social sciences	65	26.64
	Biotechnology	2	0.82
	Others	30	12.29

### Testing for Common Method Variance

Common method variance (CMV) is one of the methodological sources of measurement errors. It can potentially undermine the reliability and validity of the basic constructs and their postulated correlations in the research model. In assessing the CMV, we applied both procedural and statistical techniques to mitigate its potential impacts. The following techniques were adopted for the procedural remedies: (1) the participants were informed of the anonymity and confidentiality of the questionnaire survey; (2) honest responses were encouraged; (3) there were no right or wrong answers to survey questions; (4) ambiguous concepts were avoided; and (5) the questions were phrased concisely. It was expected that these procedural remedies diminish the method biases (Lindell and Whitney, 2001; Podsakoff et al., 2003).

According to Harman’s single-factor test, all questionnaire items measuring the entrepreneurial environment perceptions and motivations were simultaneously loaded into a factor analysis. The first principal component that emerged from the unrotated factor solution accounted for a loading of 14.54%, much lower than the 50% threshold recommended by Podsakoff and Organ (1986). Thus, we concluded that the CMV was not great enough to bias the results of this study.

### Reliability and Validity Analysis

We employed SPSS22.0 and AMOS22.0 software to analyze the reliability and validity of the data and validated the model hypotheses using a structural equation model. First, principal component analysis (PCA) was performed on the collected data. The sample data were significant at the 0.01 level, with a Kaiser–Meyer–Olkin (KMO) measure of 0.861. The analytical results revealed that the sample data were suitable for PCA. The PCA extracted six factors, which jointly explained 70.06% of the total variance. All items had a loading greater than 0.5 for their respective factors, as shown in Table 4. This indicated that the sample data had satisfactory discriminant validity and were suitable for subsequent analysis.

**TABLE 4:** Variables’ factor loading, AVE, CR, and Cronbach’s alpha.

Variable	Item	Standardized loading	AVE	CR	Cronbach’s Alpha
Government policies	GP5	0.818	0.745	0.752	0.893
	GP4	0.802			
	GP3	0.840			
	GP2	0.732			
Socio-economic conditions	SEC5	0.666	0.824	0.842	0.850
	SEC4	0.756			
	SEC3	0.736			
	SEC2	0.814			
	SEC1	0.686			
Education and training	ET5	0.511	0.798	0.752	0.838
	ET4	0.842			
	ET3	0.683			
	ET2	0.794			
	ET1	0.759			
Financial support	FS5	0.749	0.852	0.754	0.820
	FS4	0.659			
	FS3	0.834			
	FS2	0.793			
	FS1	0.507			
Non-financial support	NFS1	0.692	0.734	0.792	0.877
	NFS2	0.817			
	NFS3	0.798			
	NFS4	0.782			
	NFS5	0.747			
Entrepreneurial motivation	EM1	0.652	0.824	0.774	0.901
	EM2	0.677			
	EM3	0.602			
	EM4	0.672			
	EM5	0.545			
	EM6	0.652			
	EM7	0.742			
	EM8	0.728			
	EM9	0.697			
	EM10	0.557			
	EM11	0.695			
	EM12	0.739			

This study adopted confirmatory factor analysis (CFA) to analyze the reliability, convergent validity, and discriminant validity of the test variables. The results of the CFA are illustrated in Table 4. The average variance extracted (AVE) of each variable was greater than 0.6, which indicated that the scale had satisfactory convergent validity. The composite reliability (CR) values were all greater than 0.7, which implied that the scale had satisfactory reliability.

The results of the discriminant validity test are shown in Table 5. The square roots of the AVE for each variable were greater than the correlation coefficient between that variable and other variables. This showed that the scale had satisfactory discriminant validity.

**TABLE 5:** Variables’ square roots of AVE and correlation coefficient matrix.

	Governmental policies	Socio-economic conditions	Education and training	Financial support	Non-financial support	Entrepreneurial motivation
Government policies	0.863***					
Socio-economic conditions	0.672**	0.907***				
Education and training	0.557**	0.587**	0.893***			
Financial support	0.682**	0.647**	0.614**	0.923***		
Non-financial support	0.685**	0.735**	0.626**	0.793**	0.857***	
Entrepreneurial motivation	0.554**	0.663**	0.595**	0.546**	0.604**	0.908***

**p < 0.05; ***p < 0.01.

## Results

### Model Fit Analysis

The model fit was tested with the use of seven indices, including the degree of freedom for the chi-square (χ^2^/df), goodness-of-fit index (GFI), adjusted goodness of fit index (AGFI), normed fit index (NFI), non-normed fit index (NNFI), comparative fit index (CFI), and root mean square error of approximation (RMSEA). Table 6 lists the actual values of these model fit indices compared with their recommended and acceptable values. It was found that all of the primary indices fell within the acceptable ranges, which suggested a good fit between the theoretical model constructed in this study and the sample data.

**TABLE 6:** Model fit indices and measurement criteria.

Item	χ^2^/df	TLI	CFI	RMSEA	GFI	AGFI	NNFI
Suggested value	≤5.0	≥0.90	≥0.90	≤0.10	≥0.90	≥0.80	≥0.90
Actual value	2.458	0.827	0.841	0.000	0.942	0.842	0.923

### Hypothesis Testing

**FIGURE 2:**
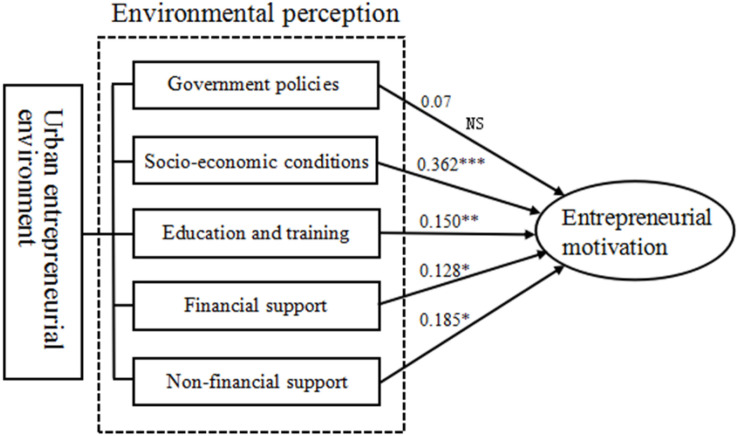
Path coefficients and hypothesis testing results of theoretical model. *p < 0.1; **p < 0.05; ***p < 0.01.

Path analysis was performed on the theoretical model using AMOS22.0 software. The path diagram is shown in Figure 2. Analysis of the posited model revealed that the path coefficient from the perceptions of socio-economic conditions to college students’ entrepreneurial motivations was 0.362, which was statistically significant (*p* < 0.01) and thus supported H2. The path coefficient from the perceptions of education and training to college students’ entrepreneurial motivations was 0.150, which was statistically significant (*p* < 0.05) and thus supported H3. The path coefficient from the perceptions of financial support availability to college students’ entrepreneurial motivations was 0.128, which was statistically significant (*p* < 0.1) and thus supported H4. The path coefficient from the perceptions of non-financial support availability to college students’ entrepreneurial motivations was 0.185, which was statistically significant (*p* < 0.1) and thus supported H5. Meanwhile, H1 was rejected because the perceptions of favorability of government policies had an insignificantly positive effect on college students’ entrepreneurial motivations.

## Discussion and Conclusion

This study primarily aimed to explore the effects of college students’ perceptions of their home cities’ entrepreneurial environments on their entrepreneurial motivations. The results revealed a significant correlation, which carried fundamental implications in four ways.

First, the perceptions of government policies’ favorability were found to affect college students’ entrepreneurial motivations positively but insignificantly. This warranted further discussions, as it was inconsistent with the findings of various scholars including Gnyawali and Fogged (1994), Stevenson and Lundstrom (2001), Garcia-Cabrera et al. (2018), and Nam and Hwansoo (2019). With continuous social progress and development, government departments are attaching increasing importance to entrepreneurship among college students and, therefore, have launched a variety of incentive policies in this regard. Nevertheless, the majority of college students are unable to either gain a sound knowledge of government policies due to their lack of social experience or fully perceive the positive effects of these policies due to limited cognitive ability. Furthermore, the policy formulation by these departments is not always oriented toward the needs of college students and may sometimes fail to fully consider the differences in policy perceptions among college students.

Second, the perceptions of socio-economic conditions played a key role in college students’ entrepreneurial motivations. This aligned with the views of scholars such as Liñán and Santos (2007), Gathungu and Baariu (2018), and Martínez-Fierro et al. (2020), all of whom posited that entrepreneurial motivation is affected by socio-economic conditions to various extents and that favorable socio-economic conditions promote the formation of entrepreneurial motivation. The results of this study also confirmed that the development of entrepreneurial motivations among college students is affected by multifaceted factors such as the growth rate of the regional economy, entrepreneurial role models, the number of successful local entrepreneurs, and the cultural atmosphere. In particular, students’ entrepreneurial preferences are determined by the local economic conditions and entrepreneurial atmosphere to a certain extent.

Third, we found that perceptions of education and training are positively correlated with college students’ entrepreneurial motivations, which was in line with the conclusions drawn by a variety of scholars such as Sirbu et al. (2015), Hou et al. (2019), and Bazan et al. (2020). This attested to the effectiveness of entrepreneurship education in higher education institutions in nurturing college students’ entrepreneurial spirits, creating a rich entrepreneurial atmosphere, and fostering students’ entrepreneurial interests and enthusiasm. Thus, education and training have positive effects on students’ entrepreneurial motivations and behaviors.

Last, but not least, the perceptions of both financial and non-financial support availability play prominent roles in college students’ entrepreneurial motivations—entrepreneurial motivation increases with increases in the magnitude of support. This finding was consistent with findings by Kristiansen and Indarti (2004), Welter (2011), Garcia-Cabrera et al. (2018), and Gathungu and Baariu (2018). As college students tend to have low financial independence, in most cases, they often have to resort to family support for sources of start-up funds. If they struggle to gain their family’s support and understanding of their self-employment career choices, their entrepreneurial plans are met with greater resistance. Difficulties in financing a business have forced a great number of aspiring college entrepreneurs to gradually lose their entrepreneurial passion and to eventually surrender to financial pressures in the real world. In addition, college students tend to take into account factors such as the entrepreneurial information resources, social networking resources, and entrepreneurial support services available in a city when choosing to start a business.

### Research Implications

The findings of this study carry a number of theoretical implications for entrepreneurial research. First, this study expanded the breadth of theoretical research on entrepreneurship. Previous studies on the relationship between an entrepreneurial environment and college students’ entrepreneurial motivations were relatively narrow. Some of them explored the role of the former in stimulating the latter based on the theory of environment, whereas others discussed how entrepreneurial motivation is influenced by pull factors in the external environment based on the theory of motivation. Nevertheless, no study has delving deeper into the types of motivational factors that incentivize different types of entrepreneurs to start their businesses (Duan et al., 2012). Research theories in cognitive psychology and entrepreneurial environment adopted in existing studies were loosely connected. A systematic summary of the effects of the perception of the entrepreneurial environment on entrepreneurial motivation was also rare, even more so when applied specifically to college students. Thus, studying the said effects among college students in the Chinese context was of profound theoretical significance.

Second, this study inspired in-depth reflections on theoretical research on entrepreneurship. It shed light on the different dimensions of the perceptions of the entrepreneurial environment on college students’ entrepreneurial motivations and, in a general sense, validated the correlations between these factors and entrepreneurial motivations. Importantly, it offered a momentous and novel finding: the perceptions of the favorability of government policies affects college students’ entrepreneurial motivations positively yet insignificantly. This was at odds with the views of some scholars such as Tan and Liu (2010), who posited that entrepreneurship education, government policies, realization of self-worth, and pressure exerted by unemployment are all important determinants of college students’ entrepreneurial motivations. This called for rethinking of the above viewpoint. Although the fundamental logic underlying this critical finding is yet to be explored, the findings of this study corroborated experience and evidence of innovative thinking in existing theoretical research, while offering implications and references for subsequent research.

Third, this study promoted a linkage across the applications of theoretical research on entrepreneurship. Centered on China’s municipal economic development challenges and dilemmas, it unraveled the real motivations for college students to pursue entrepreneurship in their home cities through an empirical survey based on the city of Wenzhou. Filling the gap in theoretical research between college students’ entrepreneurial needs and governmental efforts to improve the entrepreneurial environment offered a foothold for resolving real-life problems.

### Practical Implications

This study integrated theoretical research on the effects of college students’ perceptions of the entrepreneurial environment on their entrepreneurial motivations. Through constructing a model of factors influencing entrepreneurial motivation, it provides government departments with an impetus to optimize the urban entrepreneurial environment and incentivize college students to partake in entrepreneurial activities. This carries important implications for promoting the development of local economies.

First, when formulating incentive policies, government departments should research thoroughly the actual entrepreneurial needs of college students and give full consideration to their comprehension and incorporation into policy development. Meanwhile, they should also strengthen the proclamation and interpretation of relevant policies, thus enabling college students to fully understand their role in promoting entrepreneurship. Efforts should also be made to accelerate the development of social institutions committed to innovation and entrepreneurship, in order to ensure college students’ access to integrated services that suit their actual needs, ranging from professional counseling to project evaluations and credit guarantees.

Second, higher education institutions should adopt a changed mindset and awaken students’ entrepreneurial potential and awareness through a greater diversity of entrepreneurship education and training (Wang, 2016). Moreover, an optimized system of entrepreneurial practices for college students should be put in place by mobilizing more incubator facilities and entrepreneurial initiatives of all types. Through hands-on practice, students can experience in person the actual process of entrepreneurship and eventually lay out a firm and well-defined entrepreneurial direction.

Third, society as a whole should improve the provision of effective financial support to college students who are highly capable of innovation and entrepreneurship but in need of credit guarantee schemes. The ways to do so include innovating financing models continuously, loosening the financing requirements, simplifying the financing procedures, and increasing the upper limit of fund disbursements.

### Limitations and Future Research Opportunities

This study had several limitations, which can inform future studies. First, as quantitative analysis was adopted in this study, the accuracy of measurements might be limited by factors such as the scientific validity of the quantitative method design and the subjectivity of the respondents’ perception of the entrepreneurial environment. To perform a more comprehensive evaluation, future studies may improve the diversity of research methods by incorporating methods such as qualitative research and hybrid research. Further, this study employed convenience sampling and drew the sample entirely from a single city. This would harm the representativeness and generalizability of the results. Future studies may extend the sample to include other cities or countries and focus on inter-municipal or international comparisons. Given that cross-regional samples are informed by varied cultures, social norms, and economic backgrounds, these environmental factors may have a decisive effect on the entrepreneurial motivations within a specific sample (Raijman, 2001). In addition, the analytical results might be affected by the incomplete coverage of sample collection, which might explain why H1 was rejected in this study.

Second, there was a lack of in-depth discussion on the role individual differences play on the effects of the entrepreneurial environment on entrepreneurial motivation. An individual’s intention to stay in a city and start a business is affected by individual factors such as gender, age, and marital status (Zampetakis et al., 2015). Future studies should examine individual differences in greater depth from a demographic perspective.

Third, this study failed to disaggregate entrepreneurial motivation into separate dimensions for further analysis. This limited the investigation of environmental impacts on college students’ entrepreneurial motivations to a certain extent. Over the course of this investigation, the motivation of an entrepreneur tended to change constantly. Thus, it should be studied in a more comprehensive and complex context (Duan et al., 2012). We hope that future theoretical and empirical studies can make breakthroughs in adopting a multi-dimensional perspective that combines various factors of motivation or various stages of entrepreneurship.

## Data Availability Statement

The raw data supporting the conclusions of this article will be made available by the authors, without undue reservation.

## Ethics Statement

Written informed consent was obtained from the individual(s) for the publication of any potentially identifiable images or data included in this article.

## Author Contributions

All authors listed have made a substantial, direct and intellectual contribution to the work, and approved it for publication.

## Conflict of Interest

The authors declare that the research was conducted in the absence of any commercial or financial relationships that could be construed as a potential conflict of interest.

## References

[B1] Ahmad S. Z. Xavier S. R. (2012). Entrepreneurial environments and growth: evidence from Malaysia GEM data. *J. Chin. Entrep.* 4 50–69. 10.1108/17561391211200939

[B2] Ajzen I. (1991). The theory of planned behavior, organizational behavior and human decision processes. *J. Leisure. Res.* 50 176–211.

[B3] Aloulou W. J. (2016). Predicting entrepreneurial intentions of final year saudi university business students by applying the theory of planned behavior. *J. Small Bus. Enterp. Dev.* 23 1142–1164. 10.1108/JSBED-02-2016-0028

[B4] Bartha Z. Gubik A. S. Bereczk A. (2019). The social dimension of the entrepreneurial motivation in the central and eastern european countries. *Entrep. Bus. Econ. Rev.* 7 9–27. 10.15678/EBER.2019.070101

[B5] Bazan C. Gaultois H. Shaikh A. Gillespie K. Frederick S. Amjad A. (2020). A systematic literature review of the influence of the university’s environment and support system on the precursors of social entrepreneurial intention of students. *J. Innov. Entrep.* 9:4 10.1186/s13731-020-0116-9

[B6] Bernhofer L. Li J. (2014). Understanding the entrepreneurial intention of Chinese students: the preliminary findings of the China Project of ‘Global University Entrepreneurial Spirits Students Survey’ (GUESSS). *J. Entrep. Emerg. Econ.* 6 21–37. 10.1108/jeee-10-2013-0024

[B7] Boyd N. G. Vozikis G. S. (1994). The influence of self-efficacy on the development of entrepreneurial intentions and actions. *Entrep. Theory Pract.* 18 63–67.

[B8] Carsrud A. Brännback M. (2011). Entrepreneurial motivations: what do we still need to know? *J. Small Bus. Manag.* 49 9–26. 10.1111/j.1540-627x.2010.00312.x

[B9] Castano M. S. Mendez M. T. Galindo M. A. (2016). The Effect of Public Policies on Entrepreneurial Activity and Economic Growth. *J. Bus. Res.* 69 5280–5285. 10.1016/j.jbusres.2016.04.125

[B10] Chen Y. B. (2019). *Top 100, 1,000, and 10,000 Rankings of Chinese Enterprises with Innovation Capability. Guang Ming online.* Available online at: https://economy.gmw.cn/xinxi/2019-12/25/content_33429397.htm?s=gmwreco2 (accessed December 25, 2019).

[B11] Chen Y. J. (2013). Influence of the entrepreneurial environment on college students’ main business behaviors. *J. High. Educ. Manag.* 7 115–118. 10.13316/j.cnki.jhem.2013.03.024

[B12] Dang J. N. Wei F. (2011). A review of the research on urban entrepreneurship environment in China. *Mod. Bus.* 14 278–280. 10.14097/j.cnki.5392/2011.14.148

[B13] Davidsson P. Honig B. (2003). The role of social and human capital among nascent entrepreneurs. *J. Bus. Ventur.* 18 301–331. 10.1016/S0883-9026(02)00097-6

[B14] Djankov S. Miguel E. Qian Y. Roland G. Zhuravskaya E. (2006). Entrepreneurship: first results from Russia. *CEPR Discussion Papers* 5707 1–19. 10.2139/ssrn.641721

[B15] Duan J. Y. Wang P. Zhum Y. L. (2012). Entrepreneurial motivation research: conceptual structure, influence factors and theoretical models. *Adv. Psychol. Sci.* 20 698–704. 10.3724/sp.j.1042.2012.00698

[B16] Garcia-Cabrera A. M. Garcia-Soto M. G. Dias-Furtado J. (2018). The individual’s perception of institutional environments and entrepreneurial motivation in developing economies: evidence from Cape Verde. *S. Afr. J. Econ. Manag. Sci.* 21 1–18. 10.4102/sajems.v21i1.2377

[B17] Gathungu J. M. Baariu V. L. (2018). Competitive Strategies Entrepreneurial Orientation, External Environment and Performance of Small and Medium Enterprises in the Manufacturing Sector in Nairobi City County, Kenya. *J. Arts Hum.* 7 22–33. 10.18533/journal.v7i9.1440

[B18] Gnyawali D. R. Fogged D. S. (1994). Environments for entrepreneurship development: key dimensions and research implications. *Entrep. Theory Pract.* 18 43–62. 10.1177/104225879401800403

[B19] Gohmann S. F. (2010). Institutions, latent entrepreneurship, and self-employment: an international comparison. *Entrep. Theory Pract.* 36 295–321. 10.1111/j.1540-6520.2010.00406.x

[B20] Guo Y. Y. Chen Y. Y. Chi R. Y. (2006). Research and demonstration of evaluation method of urban entrepreneurship environment. *Sci.Technol. Prog. Policy* 2 141–145.

[B21] Hou F. Su Y. Lu M. R. Qi M. D. (2019). Model of the entrepreneurial intention of university students in the pearl river delta of China. *Front. Psychol.* 10:916. 10.3389/fpsyg.2019.00916 31114522 PMC6503157

[B22] Kibler E. (2013). Formation of entrepreneurial intentions in a regional context. *Entrep. Reg. Dev.* 25 293–323. 10.1080/08985626.2012.721008

[B23] Kristiansen S. Indarti N. (2004). Entrepreneurial intention among Indonesian and Norwegian students. *J. Enterp. Cult.* 12 55–78. 10.1142/S021849580400004X

[B24] Li H. B. Niu X. (2013). Research on the influence of entrepreneurial environment on the entrepreneurial motivation of college students. *Technol. Econ. Manag. Res.* 5 40–43.

[B25] Liñán F. Santos F. J. (2007). Does social capital affect entrepreneurial intentions? *Int. Adv. Econ. Res.* 13 443–453. 10.1007/s11294-007-9109-8

[B26] Lindell M. K. Whitney D. J. (2001). Accounting for common method variance in cross-sectional research designs. *J. Appli. Psychol.* 86 114–121. 10.1037/0021-9010.86.1.114 11302223

[B27] Locke E. A. Latham G. P. (2002). Building a practically useful theory of goal setting and task motivation: a 35-year odyssey. *Am. Psychol.* 57 705–717. 10.1037/0003-066X.57.9.705 12237980

[B28] Ma Y. (2014). The Research on Influencing Factors of University Students’ Entrepreneurial Propensity. *Chongqing Technol. Bus. Univ.* 6 1–62. 10.7666/d.Y2555746

[B29] Martínez-Fierro S. Biedma-Ferrer J. M. Ruiz-Navarro J. (2020). Impact of high-growth start-ups on entrepreneurial environment based on the level of national economic development. *Bus. Strategy Environ.* 29 1007–1020. 10.1002/bse.2413

[B30] Nam J. M. Hwansoo L. (2019). A study on the perception of entrepreneurial environment and the attitude of entrepreneurs by Asian countries: comparative analysis of China. Japan, Korea, and Singapore. *J. Entrep. Ventur. Stud.* 22 51–63.

[B31] Obschonka M. Goethner M. Silbereisen R. K. Cantner U. (2012). Social identity and the transition to entrepreneurship: the role of group identification with workplace peers. *J. Vocat. Behav.* 80 137–147. 10.1016/j.jvb.2011.05.007

[B32] Olson P. D. Bosserman D. (1984). Attributes of the entrepreneurial type. *Bus. Horiz.* 27 53–56. 10.1016/0007-6813(84)90027-2

[B33] People’s Daily (2017). *Accessed April 5, 2017.* http://www.rmlt.com.cn/2017/0405/467733.shtml?from=singlemessage&isappinstalled=0

[B34] Podsakoff P. M. MacKenzie S. B. Lee J. Y. Podsakoff N. P. (2003). Common method biases in behavioral research: a critical review of the literature and recommended remedies. *J. Appl. Psychol.* 88 879–903. 10.1037/0021-9010.88.5.879 14516251

[B35] Podsakoff P. M. Organ D. W. (1986). Self-reports in organizational research: problems and prospects. *J. Manag.* 12 531–544. 10.1177/014920638601200408

[B36] Raijman R. (2001). Determinants of entrepreneurial intentions: Mexican immigrants in Chicago. *J. Soc. Econ.* 30 393–411. 10.1016/S1053-5357(01)00101-9

[B37] Research Office of Wenzhou. Municipal People’s Government (2012). *Addressing the One-Way Exodus of Wenzhou-born College Students with Utmost Priority.*

[B38] Schwartz M. (2009). Beyond incubation: an analysis of firm survival and exit dynamics in the post-graduation period. *J. Technol. Trans.* 34 403–421. 10.1007/s10961-008-9095-x

[B39] Shane S. Locke E. A. Collins C. J. (2003). Entrepreneurial Motivation. *J. HRM Rev.* 13 257–279. 10.1016/s1053-4822(03)00017-2

[B40] Sirbu M. O. Bob C. Saseanu A. S. (2015). Entrepreneurs’ perception of their skills and the influence of education on the romanian entrepreneurial system. *Amfiteatru Econ.* 17 1213–1227.

[B41] Stevenson L. Lundstrom A. (2001). “Towards a framework for the development of entrepreneurship policy and practice,” in *Proceedings of the Annual Conference Interntionl Council Small Business*, Washington, DC.

[B42] Suzuki K. I. Kim S. H. Bae Z. T. (2002). Entrepreneurship in Japan and Silicon Valley: a comparative study. *Technovation* 22 595–606. 10.1016/s0166-4972(01)00099-2

[B43] Tan Z. H. Liu D. L. (2010). A Study on the Entrepreneurial Motivation of College Students. *Heilongjiang Res. High. Educ.* 1 129–131.

[B44] Taormina R. J. (2007). Measuring Chinese entrepreneurial motivation: personality and environmental influences. *Int. J. Entrep. Behav* 13 200–221. 10.1108/13552550710759997

[B45] Turker D. Selcuk S. S. (2009). Which factors affect entrepreneurial intention of university students? *J. Eur. Ind. Train.* 33 142–159. 10.1108/03090590910939049

[B46] Tuspark Research Institute for Innovation (2018). *Annual Report (2018): Urban Innovation and Entrepreneurship Environment Evaluation. China News.* Available online at: https://baijiahao.baidu.com/s?id=1617551282474807233&from=groupmessage&isappinstalled=0 (accessed November 19, 2018).

[B47] Wang B. Q. (2011). *Global Entrepreneurship Monitor.* Beijing: Social Sciences Academic Press, 41–52.

[B48] Wang D. J. (2016). On employment-oriented entrepreneurship education in colleges and universities. *Res. Hig. Educ. Eng.* 4 52–56.

[B49] Wang S. (2012). *Debates over the One-Way Exodus of Wenzhou College Students.* Available online at: http://www.wzrb.com.cn/article400927_256show.html

[B50] Welter F. (2011). Contextualizing entrepreneurship-conceptual challenges and ways forward. *Entrepr. Theory Pract.* 35 165–184. 10.1111/j.1540-6520.2010.00427.x

[B51] Wu W. Wang H. Zheng C. Wu Y. J. (2019). Effect of narcissism, psychopathy, and Machiavellianism on entrepreneurial intention—The mediating of entrepreneurial self-efficacy. *Front. Psychol.* 10:360. 10.3389/fpsyg.2019.00360 30846958 PMC6393355

[B52] Wu Y. J. Liu W.-J. Yuan C.-H. (2020). A mobile-based barrier-free service transportation platform for people with disabilities. *Comput. Hum. Behav.* 107:105776 10.1016/j.chb.2018.11.005

[B53] Wu Y. J. Song D. (2019). Gratifications for social media use in entrepreneurship courses: learners’ perspective. *Front. Psychol.* 10:1270. 10.3389/fpsyg.2019.01270 31214081 PMC6555126

[B54] Wu Y. J. Yuan C.-H. Pan C.-I. (2018). Entrepreneurship education: an experimental study with information and communication technology. *Sustainability* 10:691 10.3390/su10030691

[B55] Yao X. F. Wu X. Y. Long D. (2016). University students’ entrepreneurial tendency in China Effect of students’ perceived entrepreneurial Environment. *J. Entrep. Emerg. Econ.* 8 60–81. 10.1108/JEEE-03-2015-0021

[B56] Yoon N. S. (2012). The effect of potential entrepreneurial motivations on entrepreneurship and commitment to starts-up: mediating role of entrepreneurship. *J. Ind. Econ.* 25 1537–1557.

[B57] Yuan C.-H. Wu Y. J. Tsai K.-M. (2019). Supply chain innovation in scientific research collaboration. *Sustainability* 11:753 10.3390/su11030753

[B58] Zampetakis L. A. Kafetsios K. Lerakis M. Moustakis V. (2015). Investigating the role of self-construal in the formation of entrepreneurial intentions. *Front. Psychol.* 6:1085. 10.3389/fpsyg.2015.01085 26284009 PMC4518144

[B59] Zhao Q. J. Zhou B. F. Linm J. H. (2019). Influence of environmental perception on college students’ entrepreneurial intention and gender differences. *J. Hum. Agricult. Univ.* 1 89–96. 10.13331/j.cnki.jhau(ss)0.2019.01.013

[B60] Zolin R. Stuetzer M. Watson J. (2013). Challenging the female underperformance hypothesis. *Int. J. Gender. Entrep.* 5 116–129. 10.1108/17566261311328819

